# Phytochemicals and Biological Activities of *Barleria* (Acanthaceae)

**DOI:** 10.3390/plants11010082

**Published:** 2021-12-28

**Authors:** Serisha Gangaram, Yougasphree Naidoo, Yaser Hassan Dewir, Salah El-Hendawy

**Affiliations:** 1 School of Life Sciences, Westville Campus, University of KwaZulu-Natal, Private Bag X54001, Durban 4000, South Africa; serishagangaram@yahoo.com (S.G.); naidooy1@ukzn.ac.za (Y.N.); 2 Plant Production Department, College of Food and Agriculture Sciences, King Saud University, P.O. Box 2460, Riyadh 11451, Saudi Arabia; mosalah@ksu.edu.sa; 3 Department of Horticulture, Faculty of Agriculture, Kafrelsheikh University, Kafr El-Sheikh 33516, Egypt; 4 Department of Agronomy, Faculty of Agriculture, Suez Canal University, Ismailia 41522, Egypt

**Keywords:** Acanthaceae, *Barleria*, bioactive compounds, medicine

## Abstract

Plant species belonging to the family Acanthaceae are globally known to possess various medicinal properties and have cultural and economic importance in both traditional medicine and horticulture. They are important to both animals and humans and are used as food or for ornamental purposes worldwide. *Barleria* is the third largest genus in the family Acanthaceae. A few of the highly important and reported species of *Barleria* include *B. prionitis*, *B. cristata*, *B. grandiflora*, and *B. lupulina.* The flowers, leaves, stems, roots, and seed extracts of plants belonging to this genus are rich in bioactive compounds and have exhibited significant medicinal potential for the treatment of various ailments and infections. Evidence derived from several studies has demonstrated the antioxidant, antibacterial, antifungal, anti-inflammatory, anticancer, antidiabetic, antiulcer, hepatoprotective, analgesic, antiamoebic, antihelminthic, antiarthritic, antihypertensive, antiviral properties and toxicity of extracts, in addition inhibition of acetylcholinesterase activity and biosynthesis of nanoparticles, of the plant and seed extracts of species belonging to *Barleria*. Studies have reported that bioactive compounds such as flavonoids, quinones, iridoids, phenylethanoid glycosides, the immunostimulant protein “Sankaranin”, and antibiotics isolated from *Barleria* species are resposnsible for the above biological activities. Traditionally, the genus *Barleria* has significant medicinal potential; however, there is a scarcity of information on various species that are yet to be evaluated. This review provides a comprehensive report on existing literature, concerning the phytochemistry and biological activities of the genus *Barleria*.

## 1. Introduction

Traditional medicine is an ancient practice which is nearly as old as the existence of mankind. This declaration is backed by evidence obtained from studies of the older civilizations of human settlements where paleontologists discovered bunches of medicinal herbs among the fossilized remains of Neanderthal ancestors [1]. Previously, man depended solely on higher plants for medicine, and this dependence is still apparent in the present era [2,3,4,5]. Traditional preparations of plants continue to offer mankind novel remedies. Plants are rich in a diversity of secondary metabolites such as alkaloids, flavonoids, terpenoids and tannins which have been found to have antimicrobial properties [6,7,8,9]. Plant extracts have treated various infectious diseases throughout the history of mankind [10,11,12] by means of herbal preparations. These include concoctions, decoctions, infusions and teas [13]. Ancient texts of the Vedas and the Bible have described some of these traditional practices, using traditional herbs [14,15]. A great deal of conventional medicine have also originated from plant extracts, with some of the effective drugs being plant-based, such as aspirin from the bark of the willow tree [16].

**Figure 1:**
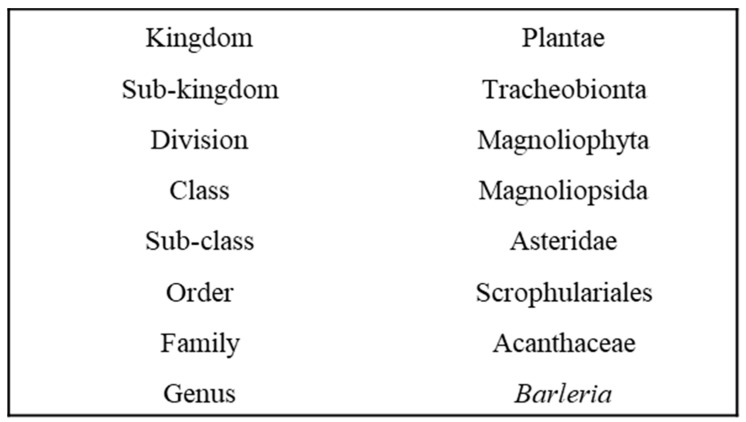
Taxonomic hierarchy of the genus *Barleria*.

**Figure 2:**
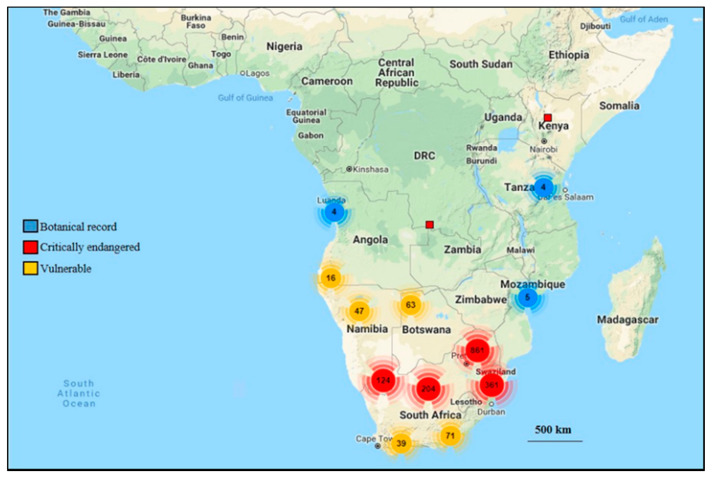
Distribution map of species of *Barleria* in Africa. Adapted from SANBI BRAHMS online (http://newposa.sanbi.org/sanbi/Explore) (accessed on 14 June 2021).

Medicines manufactured by pharmaceutical companies are largely synthetic [17]. The fear for adverse side-effects and toxicity, has brought about further scientific investigations on the potential usage of medicinal plants [18]. The increasing use of medicinal plants in various cultures has prompted scientific studies into natural products. These studies [14,15] are aimed at evaluating whether various cultures traditional practices in using natural products are supported with evidence on their pharmacological effects or if their use is simply based on folklore [19]. Due to the growing interest in the use of traditional medicine, it is essential to meet some of the concerning challenges such as: the overall lack of research, evidence of safety, efficiency and high quality of natural products, lack of patenting rights of traditional medicines and, the need to maximize and integrate natural products as possible sources of remedies in primary health care [20,21]. Various techniques have been used in extracting compounds from medicinal plants for the production of drugs. These include the isolation of compounds from plants and other natural sources, molecular modelling, synthetic and combinatorial chemistry [22]. The significance of plants as one of the natural sources of medicines can never be over-emphasized, as approximately 25% of prescribed drugs worldwide originate from plants [23]. Fifty five categorized human diseases such as cancer, parasitic and microbial infections were researched by Newman et al. [24]. It was found that 87% of medications used for treatment were derived from natural products extracted from plants. Fabricant and Farnsworth [25] showed that 122 bioactive compounds from approximately 94 plant species were consumed as clinical drugs. Knowledge of the use of plants in traditional medicine is beneficial to healers and the pharmaceutical industry. Also, the validation of the ethnomedicinal traditional practiceusing new scientific approaches can benefit a large number of individuals [26]. There is a growing need in linking the phytochemical compounds of a medicinal plant with its pharmacological activity [27]. Many plant species of Acanthaceae possess great therapeutic potential, whilst some are unexplored to-date [28]. Plant species of this family play an important role to both man and animals as they are used for food, medicine or as ornamentals [29,30,31] and contain many essential secondary metabolites, some include, alkaloids, terpenoids, tannins, quinones and flavonoids [28]. Several plant species are being utilized for their ethnomedicinal properties based on their phytocompounds they acquire, with *Barleria* (Acanthaceae) being one of such genera. The genus *Barleria* belongs to the Acanthaceae family [32] (Figure 1). The maximum representation of *Barleria* is in Africa where the diversity exists in two centers, one in tropical east Africa (approximately 80 species) and the other in southern Africa (approximately 70 species) [33]. The name *Barleria* was provided by a French botanist and Dominican monk, Jacques Barrelier (1606–1673), who dedicated his spare time to botany [34]. *Barleria* is the third largest genus in the family Acanthaceae after *Justicia* and *Ruellia* [35,36,37], and it is the most species-rich genus in *Barlerieae* [38]. This genus includes approximately 300 species of shrubs and herbs that are distributed in the subtropical and tropical regions of the world [39,40,41,42,43]. The members of this genus have originated from the Far East of Japan, through southern Asia, Arabia, India, Kenya, Tanzania, Angola, Democratic Republic of the Congo, Namibia, Botswana, Mozambique, southern Africa, and Madagascar to as far West of Central America and Mexico [33,42]. *Barleria* is predominantly an “Old World genus” (a term used in the West to refer to Africa, Asia, and Europe), with its maximum species diversity being present in east tropical Africa followed by South Africa [33]. The distribution of *Barleria* throughout Africa is illustrated in Figure 2. In southern Africa, there are 70 species of *Barleria* of which approximately 65% are endemic to the region [43,44].

*Barleria* can be easily distinguished from other genera within Acanthaceae based on the following three features: (i) a four-partite calyx consisting of two outer large segments and two smaller inner segments, (ii) globular, honeycombed pollen, and (iii) the prevalence of double cystoliths located in the epidermal cells [33,40,45]. The fruits of *Barleria* are hygrochastic [46], implying that the opening of the fruit is initiated by moisture or water [47,48]. In *Barleria*, the cystoliths are always double and lie in two adjacent cells. These structures are scattered over the leaf lamina and often lie parallel on the midrib [49,50,51]. Several species of *Barleria* are known for their medicinal or ornamental values [37,52,53]. There have been studies reviewing the traditional, phytochemical and pharmacological properties of specific species of *Barleria* (e.g., *B. lupulina* and *B. prionitis*) [54,55,56], however none have comprehensively reported on species within *Barleria* using exisiting literature in a clear and concise manner. Therefore, this review is intended to elaborate on only the important and extensively studied species belonging to the genus *Barleria*, with an emphasis on their biological activities that have been published.

## 2. Phytochemical Evaluation of *Barleria*

### 2.1. Phytochemicals Isolated from Barleria

Plants possess the ability to synthesize various secondary metabolites, among which at least 57182 have already been isolated [57]. It is important to determine the relationship between the phytochemical compounds of a medicinal plant and its pharmacological activity. A few of the highly important species of *Barleria* include *B. prionitis*, *B. cristata*, *B. grandiflora*, and *B. lupulina* [42,52]. Several authors have reported that species belonging to this genus exert biological effects, including antibacterial, antifungal, anti-inflammatory, anticancer, antidiabetic, antiulcer, hepatoprotective, analgesic, antiamoebic, antihelminthic, antiarthritic, antihypertensive, and antiviral activities and inhibition of acetylcholinesterase activity [58,59,60,61,62,63,64,65]. Studies have reported that bioactive compounds such as flavonoids, quinones, iridoids, phenylethanoid glycosides, immunostimulant protein “sankaranin,” and antibiotics that are isolated from *Barleria* species are responsible for the abovementioned biological activities [66,67,68,69,70]. Jäger et al. [71] suggested that when bioactive compounds are detected in a plant species, it is possible that numerous species of the same genus contain active compounds of a similar nature. It has been reported that *Barleria* consists of various secondary metabolites that have been primarily isolated from the flowers, leaves, stems, roots, and seeds of the plant (Table 1). The important phytochemical compounds isolated from *Barleria* are iridoids, phenolic acids, phenylethanoid glycosides lignans, flavonoids, and phytosterols (Table 1).

#### 2.1.1. Iridoids

Chemical compounds such as iridoids are monoterpenes that are biosynthesized from isoprene and are also identified to be precursors in the biosynthesis of alkaloids [96,97,98,99]. Like glycosides, iridoids are generally found in various medicinal plants and are most often bound to glucose [96,97]. Iridoids that are isolated and purified exhibit a broad spectrum of bioactivities, including antihepatotoxic, choleretic, hypoglycemic, cardiovascular, anti-inflammatory, antimutagenic, antitumor, antiviral, and analgesic activities [96,97]. Some important iridoid medicinal compounds found in *Barleria* include barlerin, shanzhiside methyl ester, ipolamiide, acetylbarlerin, phlorigidoside, lupulinoside, 7-methoxydiderroside, isoverbascoside, decaffeoylverbascoside, and 10-*O*-*trans*-coumaroyl-eranthemoside (Table 1).

#### 2.1.2. Phenolic Compounds (Acids/Glycosides/Lignans/Neolignans)

Phenolic acids are natural compounds that are prevalent throughout the plant kingdom. They are involved in a variety of biological activities such as antimicrobial, anti-inflammatory, antioxidant, antidiabetic, hepatoprotective, and anticancer properties [98,100,101,102,103]. Phenolic acids can be categorized into hyrdoxybenzoic acids that contain seven carbon atoms and cinnamic acids that contain nine carbon atoms (C6-C3). Phenolic compounds derived from plants are different in their molecular structure and are typically characterized by their hydroxylated aromatic rings [104]. In several plants, phenolic compounds are polymerized into large molecules such as lignins and proanthocyanidins (condensed tannins). The antioxidant capacity of phenolic compounds has attracted the attention of researchers, as these compounds can reduce the risk of developing several diseases and protect the human body from free superoxide radicals [105]. The important phenolic acids found in *Barleria* include *p*-hydroxycinnamic acid, *p*-coumaric acid, α-tocopherol, melilotic acid, syringic acid, vanillic acid, and *p*-hydroxybenzoic acid (Table 1). The aromatic compound 4-hydroxy-*trans*-cinnamate derivative found in *Barleria* was isolated from *B. cristata* [70]. The phenolic glycosides found in this genus are barlerisides A and B (Table 1).

Lignans and neolignans are a group of large, naturally occurring phenols that are derived from the shikimic acid biosynthetic pathway and have a wide distribution within the plant kingdom [106]. Both class of compounds exhibit dimeric structures that are formed by a β-linkage between the two phenyl propane units and an altered degree of oxidation in the side chain [106]. One of the major ecological functions of lignans is protecting the plants that synthesize them against herbivores and microorganisms [107].

One lignan glucoside, (+)-lyoniresinol 3α-*O*-β-glucopyranoside, was isolated from the aerial plant parts of *B. lupulina* (Table 1). In *B. acanthoides*, one type of neolignan diglycoside, barlericin, was isolated from the entire plant (Table 1).

#### 2.1.3. Flavonoids

Flavonoids are present in the leaves, flowers, and pollen of several plants and comprise a group of polyphenolic compounds [108]. Flavones, flavanones, flavonols, isoflavones, and anthocyanins are the major classes of flavonoids that have been reported to possess a broad spectrum of biological and therapeutic activities [109]. Studies have also reported that flavonoids or flavonoid-rich extracts exhibit antioxidant, anti-inflammatory, and antimicrobial activities [110,111,112,113,114]. Flavonoids play a vital role in inhibiting the activity of important enzymes in mitochondrial respiration and in protection against heart diseases [115]. This compound has the potential to prevent early stages of cancer due to its ability to scavenge free radicals [116]. A total of 14 flavonoids (Table 1) have been isolated from various plant parts of *Barleria*, including 6-*O*-α-l-rhamnopyranoside-3,7,3′-*O*-trimethylated-8-hydroxyquercetin, 6-*O*-α-l-rhamnopyranoside quercetagetin, 3-*O*-methylquercetin, gossypetin 8-methyl ether, quercetagetin, tamarixetin, gossypetin, quercetin, luteolin, 7-*O*-methylluteolin, apigenin 7-*O*-β-d-glucoside, 6-hydroxyflavone, apigenin 7-*O*-α-l-rhamnosyl-(1→6)-*O*-β-d-glucoside, scutellarin, and luteolin-7-*O*-β-d-glucoside.

#### 2.1.4. Terpenoids

Terpenoids are the most frequent and structurally diverse organic compounds that are derived from five-carbon isoprene units [117]. Terpenoids are classified based on the number of isoprene units, such as hemiterpenoids (C5), monoterpenoids (C10), sesquiterpenoids (C15), diterpenoids (C20), sesterterpenoids (C25), and triterpenoids (C30) [117,118]. A large number of terpenoids are of plant origin and have several biological roles in higher plants [119,120]. In addition, four terpenoid compounds, oleanolic acid, balarenone, pipataline, and lupeol, were isolated from the aerial parts and the entire plant of *Barleria* (Table 1). The terpenoids isolated from plant extracts are known to possess antiviral, antifungal, antibacterial, anti-inflammatory, antihyperglycemic, anticancer, and insecticidal properties [121].

#### 2.1.5. Phytosterols (Terpernoids)

Phytosterols are an important family of lipids that are typically found in plants and fungi and are essential to humans because of their nutritional and medicinal values. Phytosterols also function as precursors in the production of essential bioactive compounds such as steroidal glycoalkaloids, steroidal saponins, brassinosteroids, and phytoecdysteroids [122]. They are grouped into 24-ethylsterols and 24-methylsterols [123]. Some examples of 4-desmethylsterols that are abundantly found in most of the plants are campesterol, sitosterol, and stigmasterol [124]. Only two isolated phytosterols have been reported in *B. prionitis*, viz., 13,14-seco-stigmasta-5,14-diene-3-β–ol and β-sitosterol (Table 1).

#### 2.1.6. Phenylethanoid Glycosides

Phenylethanoid glycosides are a group of aqueous-soluble compounds, with majority of them have been isolated from medicinal plants [125,126]. The general structure of phenylethanoid glycosides has one glucopyranoside unit linked to the phenethyl alcohol. This compound has chemotaxonomic relevance being considered with one additional chemotaxonomic marker in several families of Asterids, in particular when in co-occurrence with iridoids [127]. Phenylethanoid glycosides have been described to contain novel structures with diverse bioactivities [128,129]. Five phenylethanoid glycosides, viz., acteoside (synonym verbascoside), desrhamnosyl acteoside, poliumoside, forsythoside and barlerinoside, have been isolated from several species of *Barleria*. However, verbascoside (synonym acetoside), was isolated from *B. acanthoides*, *B. prionitis*, and *B. strigosa* (Table 1).

## 3. Biological Activities of Extracts, Fractions, and Isolated Compounds from *Barleria*

### 3.1. Antioxidant Properties

Antioxidants are defined as substances that inhibit or delay oxidative damage to a specific molecule [130]. Oxidative stress is a key contributor to various chronic diseases [131]. It implies a disruption in the imbalance between reactive oxygen species (ROS), free radicals (FR), and the endogenous antioxidant defense mechanisms [132]. When antioxidant molecules encounter single FR, they neutralize them by donating one of their own electrons, which in turn ends the carbon-stealing reaction [133,134]. The antioxidant defense mechanisms in plants are enzymatic and nonenzymatic. The enzymatic defense mechanism includes catalase (CAT), peroxidase (POX), and superoxide dismutase. These antioxidants effectively mitigate cell damage against ROS. The nonenzymatic antioxidant mechanism consists of carotenoids, vitamin C, vitamin E, and flavonoids [135,136,137]. There is substantial evidence indicating that FR cause oxidative damage to biomolecules (nucleic acids, lipids, and proteins), which eventually results in aging, atherosclerosis, diabetes mellitus, cancer, acquired immunodeficiency syndrome (AIDS), inflammation, and various degenerative diseases in humans [138]. Plants are a source of natural antioxidants, including phenols, flavonoids, ascorbic acid, and carotenoids. Ascorbic acid and β-carotene are one of the widely used antioxidants [139].

The reported antioxidant properties of various extracts and isolated compounds of *Barleria* are summarized in Table 2. Various methods have been used to evaluate the antioxidant activities of aqueous, acetone, ethanol, ethyl acetate, hydroalcoholic and methanolextracts and those of the isolated compounds barlerisides A and B, shanzhiside methyl ester, 6-*O*-*trans*-*p*-coumaroyl-8-*O*-acetylshanzhiside, methyl ester, barlerin, acetylbarlerin, 7-methoxydiderroside, and lupulinoside. Antioxidant activity was observed and reported in all plant extracts by several researchers using various assays. The most frequently investigated species within the genus is *B. prionitis*. Amoo et al. [98] examined the methanolic extracts of the different parts of *B. prionitis* using the 1,1-diphenyl-2-picrylhydrazyl (DPPH) scavenging assay and reported that the extracts exhibited free radical scavenging activity, with the EC_50_ values varying from 6.65 to 12 µg/mL. In addition, they evaluated the ferric reducing antioxidant power and the β-carotene bleaching rate of the extracts and found that the extracts reduced the ferric ion complex to the ferrous form and decreased the carotenoid bleaching rate. The findings of that study suggest the occurrence of antioxidant compounds in the methanol extracts, which are capable of donating electrons and hydrogen atoms in their reactions [98]. Moreover, Jaiswal et al. [140] evaluated the β-carotene bleaching potential, and the hydroxyl radical scavenging activity of the ethanolic extracts of *B. prionitis*. They found the highest β-carotene bleaching rate of 79.20% ± 1.26% compared to those of flower (62.16% ± 2.56%) and stem (48.31% ± 1.960%) extracts. The leaf extract exhibited good free radical scavenging activities compared to the other plant extracts, with the IC_50_ values being 336.15 ± 7.21 μg/mL for DPPH and 568.65 ± 6.11 μg/mL for the hydroxyl radical. Quercetin was used as the standard for DPPH and hydroxyl radicial with IC_50_ values of 0.021 ± 0.004 ug/mL and 0.072 ± 0.007 ug/mL, respectively. Various species within the genus *Barleria* exhibit excellent antioxidant properties. Therefore, the antioxidants found in *Barleria* plant extracts exhibiting free radical scavenging activities may play an important role as therapeutic agents in numerous diseases that are related to oxidative stress [141].

### 3.2. Antibacterial Activity

Infectious diseases are a serious concern in Africa [164]. One of the primary causes of ill health and death are bacterial infections [165,166,167]. The extensive use of antibiotics to treat bacterial infections has encouraged researchers to screen medicinal plants for antibacterial activity [168]. Plant species belonging to the genus *Barleria* (Acanthaceae) are known to exhibit exceptional antibacterial properties. Several studies have demonstrated the antibacterial activity of extracts and isolated compounds of *Barleria* (Table 3). The antibacterial activity of the various plant extracts has been evaluated against the Gram-positive bacteria *Bacillus cereus*, *Bacillus pumilus*, *Bacillus* sp., *Bacillus subtilis*, *Enterococcus faecalis*, *Lactobacillus acidophilus*, *Lactobacillus rhamnosus*, *Lactobacillus sporogenes*, *Micrococcus luteus*, *Staphylococcus aureus*, *Staphylococcus epidermidis*, *Streptococcus mutans*, and *Streptococcus pyogenes* and the Gram-negative bacteria *Comamonas acidovorans*, *Citrobacter* sp., *Enterobacter aerogenes*, *Escherichia coli*, *Klebsiella pneumoniae*, *Pseudomonas aeruginosa*, *Pseudomonas fluorescens*, *Proteus mirabilis*, *Providencia* sp., *Pseudomonas* sp, *Proteus vulgaris*, *Salmonella paratyphi*, *Salmonella typhi*, *Shigella dysenteriae*, *Serratia marcescens*, *Vibrio cholera*, and *Xanthomonas oryzae*. The most commonly investigated species for the antibacterial activity within the genus is *B. prionitis.* Amoo et al. [66] examined the minimum inhibitory concentration (MIC) of the petroleum ether, dichloromethane, and ethanol extracts of *B. prionitis*. Neomycin was used as a positive control against each bacterium.

These authors and found that these extracts exhibited a broad spectrum of antibacterial activity. The MIC values ranged from 781 to 3125 μg/mLfor *B. subtilis*, *S. aureus*, *E. coli*, and *K. pneumoniae*. These MIC values were compared to neomycin which ranged from 1000 to 1563 μg/mL. MIC values were (>100 μg/mL) from the tested extracts and were considered moderately effective when compared to neomycin, while others displayed low antibacterial activity. Findings from this study demonstrated the potential of *B. prionitis* as an antibacterial agent, while further studies are necessary. Furthermore, Aneja et al. [169] evaluated the antibacterial activity of the acetone, ethanol, methanol, and water extracts of *B. prionitis* bark. Ciprofloxacin served as the positive control. Their study results suggested that the methanolic bark extract (100 μg/mL) was the most effective against all four oral bacteria with varying inhibition zones (*S. mutans* (15.65 ± 0.57 mm)*, S. aureus* (16.32 ± 0.57 mm), *Pseudomonas* sp. (19.32 ± 0.57 mm), and *Bacillus* sp. (28.65 ± 0.57 mm). Zones of inhibition values of the tested extract were compared to ciprofloxacin (100 μg/mL) which ranged from 27.32 ± 0.57 mm to 29.65 ± 0.57 mm. Therefore, the methanolic bark extract displayed promising antibacterial activity when compared to ciprofloxacin. Statistical analyses on the antibacterial activity of crude extracts are lacking in both studies, this should be further explored.

### 3.3. Antifungal Activity

Opportunistic fungal infections can become fatal to individuals with immunocompromised conditions [187], in particular those with cancer [188] and HIV/AIDS [189]. Management of these infections has become complex due to the limited number of cost effective antifungal agents, toxicity of the accessible agents, relapse of infections, and resistance to these commonly used agents [190,191]. Consequently, it has become critical to explore naturally occurring antifungal agents. *Barleria*, being one of such genera, has exhibited excellent antifungal properties. Numerous studies have validated the antifungal activity of the extracts and fractions of *Barleria* (Table 4). Aneja et al. [169] evaluated the antifungal activity of acetone, ethanol, and methanolic extracts of *B. prionitis* and found that the extracts significantly reduced the growth of fungi, with the maximum zone of inhibition being observed for *Candida albicans* (100 μg/mL) strain 1 (13.65 ± 0.57, 12.94 ± 1, and 15.31 ± 0.57 mm), *C. albicans* (100 μg/mL) strain 2 (16 ± 0, 11.31 ± 0.57, and 16.96 ± 1 mm), and *Saccharomyces cerevisiae* (100 μg/mL) (11.64 ± 0.57, 11.31 ± 0.57, and 13.95 ± 1 mm). Amphotericin-B (100 μg/mL) served as the positive control, with inhibition zones ranging from 11.94 ± 1 mm to 13 ± 0. Results of the tested extracts were similar to/ or higher then amphotericin-B, thus displaying siginificant antifungal activity.

Furthermore, Amoo et al. [98] demonstrated the fungicidal activity of extracts derived from different parts of *B. prionitis* against *C. albicans*. Amphotericin B was used as a positive control in this study. They reported a minimum fungicidal concentration range of 4700–6300 μg/mL for the extracts of stems and roots. Minimum fungicidal concentration (MFC) for amphotericin B was 0.193 µg/mL. Therefore, the tested extracts displayed low antifungal activity (<100 µg/mL) when compared to the positive control. Statistical analyses on the antifungal activity of crude extracts are lacking in both studies, this should be further explored.

### 3.4. Anti-Inflammatory Activity

Several deteriorating diseases such as shoulder tendonitis, gouty arthritis, rheumatoid arthritis, polymyalgia rheumatica, asthma, cancer, heart disease, and inflammatory bowel disease are related to inflammatory processes [93,198,199]. Scientific researchers and pharmaceutical companies have been showing a growing interest in identifying novel anti-inflammatory compounds in medicinal plants. This can potentially lead to the production of novel drugs in treating pain-related ailments with no side effects [200]. Several studies have validated the anti-inflammatory activity of the extracts and fractions of *Barleria* (Table 5). Amoo et al. [66] evaluated the anti-inflammatory activity of petroleum ether, dichloromethane, and ethanolic extracts using cyclooxygenase (COX)-1 and COX-2 assays. The positive control used was indomethacin, with a concentration of 5µM for COX-1 and 200µM for COX-2. They reported that petroleum ether extracts (leaf (72.5% ± 1.26%) and root (77.2% ± 1.41%) and dichloromethane extracts (leaf (79.7% ± 1.55%)) of *B. prionitis* exhibited promising activity (>70%) in COX-1 assay. Indomethacin inhibited prostaglandin synthesis in COX-1 assay with a value of 63.4 ± 1.98%. Moreover, in COX-2 assays, the root, petroleum ether (78.5% ± 1.90%), and dichloromethane extracts (70.4% ± 1.80%) of *B. prionitis* demonstrated the best activity (>70%). The nonpolar extracts (petroleum ether and dichloromethane) exhibited greater activity than ethanolic extract. Additionally, for COX-2 assay, indomethacin inhibited prostaglandin synthesis (73.6 ± 1.47%). Statistical analysis showed extracts had significantly different activity (*p* < 0.05). Overall, extracts, to some degree, presented good anti-inflammatory activity. Cos et al. [192] reported that compounds that are strong inhibitors of enzymes fail in vitro to settle against the entire organism, as their passage toward the cell membrane is restricted. In addition, Zschocke and Van Staden [201] explained that the activity exhibited by nonpolar extracts is of significant interest because the lipophilic compounds extracted from these solvents exhibit better resorption through the cell membrane. Overall, their study results demonstrated that the anti-inflammatory activity of these extracts is related to their inhibition of cyclooxygenase enzymes, following the inhibition of prostaglandin synthesis. Singh et al. [202] examined the anti-inflammatory activity of methanol–aqeuous fractions (TAF) of *B. prionitis* on different acute and chronic animal test models. They observed that the iridoid-enriched fraction demonstrated activity against carrageenan-, histamine-, and dextran-induced inflammation models. Ibuprofen served as a standard for authenticity of the experiment. Marked inhibitory effect was exhibited by TAF in a dose-dependent manner on carrageenan-induced edema (normal rats), with the ED_50_ values being 89.70 and 143.51 mg/kg (11.93–44.56%) in adrenalectomized rats. Ibuprofen exhibited inhibition with a value of 54.03 ± 2.51%. The oral administration of TAF inhibited histamine- and dextran-induced edema, with the ED_50_ values being 333.52 mg/kg (12.16–36.14%) and 467.19 mg/kg (12.35–34.05%), respectively. Additionally, the standard drug displayed inhibition with a value of 41.23 ± 2.55%. Therefore, the tested extract displayed promising anti-inflammatory activity when compared to the standard drug.

### 3.5. Anticancer Activity

Worldwide, cancer has been considered as the most critical disease in humans due to its high morbidity and mortality rates [210]. Radiotherapy, surgery, and chemotherapyare the primary therapies used to treat cancer. Although these therapies have saved the lives of several patients with cancer, the severe side effects and the high relapse rates have rendered them only moderately effective to control and in certain cases cure cancers. Therefore, there is an urgent need to develop more diverse and effective therapies from several sources [210]. Compared with synthetic chemotherapeutic drugs, natural chemicals derived from plants are relatively less toxic and possess high target specificity. Therefore, the potential usage of medicinal plants as anticancer drugs is important. In this regard, *Barleria* has demonstrated significant potential for anticancer activity, with several studies reporting the potent activity of extracts and isolated compounds against tumor cell lines (Table 6). In addition to *B. prionitis*, *B. cristata* and *B. grandiflora* have been frequently reported to exhibit potent anticancer activities (Table 6). El-Halawany et al. [211] examined the anticancer effects of phenolic compounds (verbascoside, isoverbascoside, dimethoxyverbascoside, *p*-hydroxybenzoic acid, and apigenin-7-*O*-glucoside) isolated from *B. cristata*. They found that preliminary treatment of Hepa-1c1c7 cells with 3.125 μM of the tested isolated compounds inhibited the cytotoxic effect caused by menadione. Sulforaphane (5 μM) served as the positive vehicle control. Among the tested compounds, the best results were observed for isoverbascoside, which potently induced the activity of the enzyme in a dose-dependent manner. Isoverbascoside exhibited the strongest effect in protecting Hepa-1c1c7 cells against the toxicity of menadione (quinone substrate for NQO1), causing an 8.8-fold induction of NQO1 activity at 25 μM (compared with vehicle control activity level). The anticancer activity of the various phenolic compounds and controls used in this study is lacking statistical analyses, this may be a limitation which should be further explored. In addition, Manglani et al. [212] evaluated the anticancer activity of the leaf extracts of *B. grandiflora* on various normal and cancerous cell lines such as human lung cancer cells (A-549), Dalton’s lymphoma ascites (DLA tumor cells), and African green monkey kidney (Vero) normal cells. Standard drug 5-Flurouracil (20 mg/kg) was the positive control in this study. They found that alcoholic leaf extracts exhibited cytotoxic effects against A-549 (IC_50_ values (alcoholic extract 143.4 μg/mL, aqueous extract 210.8 μg/mL) and DLA (IC_50_ values (alcoholic extract 137.2 μg/mL, aqueous extract 217.8 μg/mL). The anti tumor activity of the alcoholic extract against DLA tumor bearing mice was assessed in vivo. The tumor volume, and viable cell count were significantly (*p* < 0.01) increased and non viable cell count had significantly (*p* < 0.01) declined in DLA control animals, when compared with normal control animals. The administration of the alcoholic extract in vivo, at 200 and 400 mg/kg significantly (*p* < 0.01) decreased the tumor volume and viable cell count. Overall, the alcoholic extract was potent to the Vero cell line, witha IC_50_ value of148.7 μg/mL, while the aqueous was less potent, with a IC_50_ value of 52.6 μg/mL. Their study showed that the alcoholic extracts were less toxic to human cells and exhibited significant in vitro and in vivo antitumor activity against DLA cells.

### 3.6. Antidiabetic Activity

Based on folkloric claims, people with diabetes have been treated orally with various medicinal plants or their extracts since ancient times [216]. Hypoglycemic synthetic agents can produce severe side effects, including liver and kidney function disturbances and hematological coma [217]. Therefore, the search for more safe and effective antidiabetic agents in plants has continued to be a critical area for research. *Barleria* species have also demonstrated antidiabetic activities as shown in Table 7. According to Singh et al. [218], oral administration of ethanolic seed extract (200 mg/kg) from *B. cristata* for 7 days decreased blood glucose levels in a model of alloxan-induced diabetes in rats. The control group received normal saline only. Statistical analysis is lacking in this study. Therefore, further studies should evaluate the active compounds responsible for the anti-diabetic displayed in *B. cristata.* Furthermore, Vasanth et al. [147] investigated the ethanol and petroleum ether leaf extracts of *B. cristata* for their antidiabetic activity and found that both extracts exhibited dose-dependent increases in the inhibitory activities of α-glucosidase (inhibition: ethanol extract 47% and petroleum ether extract 44%) and α-amylase (inhibition: ethanol extract 67% and petroleum ether extract 61%) at a concentration of 100 μL. Butylated hydroxytoluene served as the positive control. Overall, the best results were obtained with ethanol extracts that demonstrated the maximum in vitro antidiabetic activity compared with petroleum ether extract. It is recommended that further investigations should evaluate other compounds as positive controls (i.e., acarbose). Reema and Pradeep [219] reported about the antidiabetic properties of *B. prionitis*. The control group received distilled water. They observed a reduction in glycosylated hemoglobin (*p* < 0.01) and blood glucose (*p* < 0.01) levels in alloxan-induced diabetic rats treated with the ethanolic leaf extract. A further observation in their study was an increase in liver glycogen and serum insulin levels but a decrease in body weight. In experimental animals, the root ethanolic extract (200 mg/dL) exhibited a moderate but nonsignificant antidiabetic activity. Further studies should elucidate its mechanism in detail. The above-described results thus confirm the antidiabetic potential of the various species of *Barleria*, however further studies should be conducted to for further validation.

### 3.7. Antiulcer Activity

Gastric hyperacidity is a common problem that affects millions of individuals worldwide due to an imbalance between protective and aggressive factors [226]. Peptic ulcers are generally treated using proton pump inhibitors, H_2_ receptor antagonists, and antimuscarinics. However, the majority of these agents produce adverse effects such as impotence, arrhythmia, gynecomastia, hypersensitivity, and hematopoietic disorders [227]. Therefore, it is crucial to explore plants containing natural antiulcer and antioxidant compounds that can be used as safer treatment alternatives with less side effects. Several studies have demonstrated the antiulcer activity of extracts of *Barleria* (Table 8). Kumar and Singh [52] investigated the antiulcer activity of the methanolic leaf extracts of *B. prionitis*. They reported a statistically significant reduction (*p* = 0.05) of ulcer index in the treated animals in comparison with control groups in both models. Ranitidine (50 mg/kg) served as the positive control in this study exhibited significant protection, *p* ˂ 0.01. Substantial changes were observed only in the total acidity at a dose of 500 mg/kg, and changes were significant in the levels of aspartate aminotransferase (AST) and alanine aminotransferase (ALT) at both doses in the ethanol-induced gastric ulcer model. Further studies are required to isolate compounds from these extracts and elucidate their mechanism of action. Jaiswal et al. [228] examined the gastroprotective effect of iridoid fractions obtained from the leaves of *B. prionitis* against various gastric ulcer models in rats. They observed that the fractions exhibited a dose-dependent ulcer-protective effect in ulcer models induced by pylorus ligation (PL) (18.67–66.26% protection), aspirin (24.65–63.25% protection), cold-restraint stress (CRS) (20.77–59.42% protection), and ethanol (16.93–77.04% protection). Ranitidine and sucralfate were used as the positive control.The iridoid fractions derived from *B. prionitis* demonstrated antiulcerogenic properties (200 mg·kg^−1^) by decreasing the acid-pepsin secretions in rat models of gastric ulcer [228]. The fractions reduced the ulcer index by significantly decreasing the lipid peroxidation product (*p* < 0.01–0.001) in comparison to the control, and superoxide dismutase activity (*p* < 0.01–0.001) and increasing the catalase activity in the CRS-induced model.

### 3.8. Hepatoprotective Activity

Liver diseases (acute and chronic) are a global concern [231], and their treatment is difficult to achieve because none of the available drugs have been effective in stimulating liver function or aiding the liver to regenerate hepatic cells [232,233]. In addition, hepatotoxic chemicals cause damage to liver cells by accelerating lipid peroxidation and other oxidative injuries [234,235,236,237,238]. Hence, due to increasing incidences of chemically induced hepatotoxicity, there is a demand for safe protective agents [233]. Conseqeuntly, it is essential to explore alternative drugs from plant sources that are safe and efficient in treating liver diseases. Therefore, several medicinal plants, especially within the genus *Barleria*, have been screened for hepatoprotective activity by various researchers (Table 9). For instance, Balaji et al. [239] investigated the hepatoprotective activity of the ethanolic leaf extracts of *B. cristata* against CCl_4_ (0.7 mL/kg, i.p)-induced hepatic damage in Wistar albino rats (at dose levels of 100–200 mg/kg). Silymarin was administered as the positive control in this study. The ethanolic extract significantly (*p* < 0.001) decreased the serum levels of specific liver enzymes such as alanine aminotransferase, aspartate aminotransferase, and alkaline phosphatase and total protein, total bilirubin, triglyceride, and cholesterol levels. They used a known hepatoprotective drug, silymarin (25 mg/kg), for comparison that displayed significant activity (*p* < 0.001). The ethanolic extract did not cause any mortality in the Wistar rats (up to a dose level of 200 mg/kg). Overall, their study results indicated that the ethanolic extract exhibited hepatoprotective properties, which may be due to the presence of flavonoids and alkaloids [239].

Singh et al. [240] also evaluated the iridoid-enriched fractions obtained from the ethanol–aqueous leaf and stem extracts of *B. prionitis* for hepatoprotective activity in Charles Foster rats and Swiss albino mice. These fractions exhibited hepatoprotective activity in several chronic and acute animal test models. Silymarin; Liv-52 and stimuliv were used as positive controls to compare the results. After a single dose of drug administration, the oral LD_50_ value was found to be >3000 mg/kg, with no signs of deformities or mortality (for a duration of 15 days). However, the intraperitoneal LD_50_ was found to be 2530 ± 87 mg/kg in mice. Although the maximum tolerance dose is high, the safety evaluation of animal models displayed no signs of abnormalities or any mortality. Statistical analysis showed a significant difference between control and the drug treated groups. The extracts provided significant hepatoprotection against hepatotoxicity induced by galactosamine, carbon tetrachloride, and paracetamol. Overall, their study reported significant and concentration-dependent hepatoprotective activity of the iridoid-enriched fractions, as most of the altered hepatic parameters in experimental rodents (liver damage) were reversed. Further studies should be conducted to assess the extracts safety. Hence, extracts derived from *Barleria* have hepatoprotective properties that can serve as an effective treatment for acute hepatic diseases.

### 3.9. Analgesic Activity

Unbearable and long-term pain is one of the primary causes for poor quality of life, and therefore, several researchers are investigating the mechanisms and causes of pain and are exploring novel drugs in plants to reduce pain with less to no side effects. Although analgesic drugs are available and efficient in reducing pain, their repetitive application can cause several side effects such as tolerance and physical dependence [244,245]. Regarding the genus *Barleria*, the literature reports only one study conducted by Begum et al. [246], who investigated the effects of ethanol, chloroform, and petroleum ether extracts of the aerial parts of *B. prionitis* in Swiss albino mice at doses of 200 and 400 mg/kg. Overall, their study indicated that all the three extracts demonstrated significant analgesic effects in mice, with inhibition proportions of 30.36%, 59.40%, and 33.70% when tested at 400 mg/kg.

### 3.10. Antiamoebic Activity

A common intestinal infection occurring in humans in developing countries is amoebiasis, which is caused by the protozoan parasite *Entamoeba histolytica*. Trophozoites of *E. histolytica* invade the intestinal mucosa, resulting in dysentery, and thereafter sporadically migrate to the liver, triggering abscesses [247,248]. Although the drugs used in the treatment of amoebiasis are effective, they induce various side effects such as nausea, stomatitis, gastrointestinal discomfort, and vomiting [249,250,251,252,253]. Therefore, it is essential to identify new compounds in plants possessing antiamoebic activity that are safe for human usage. Till date, only one study has been conducted on the antiamoebic activity of a species of *Barleria*. Sawangiaroen et al. [254] evaluated the antiamoebic activities of the chloroform leaf and stem extracts of *B. lupulina* at a concentration of 1000 μg/mL against the *E. histolytica* strain (HM1:IMSS). Metronidazole served as the positive control. They observed that the chloroform extract derived from *B. lupulina* stem exhibited the best antiamoebic activity (IC_50_ 78.5 μg/mL) against *E. histolytica* then when compared to the leaf extract. The stem extract was classified as active, with an IC_50_ value of < 100 μg/mL [254]. The IC_50_ of a standard drug, metronidazole, was 1.1 μg/mL.

### 3.11. Antihelminthic Activity

Helminths are parasitic worms that are infectious to humans in developing countries [255]. These worms reside in the gastrointestinal tract and can burrow into the liver and other organs. Infected individuals excrete helminth eggs in their fecal matter, causing the contamination of soil in areas with poor sanitation [256]. The drugs used to treat these infections have common side effects such as vomiting, nausea, abdominal pain, allergic reactions, expulsion of ascaris from mouth or nose, body ache, and fever [257]. Consequently, the search for plants exhibiting antihelminthic activity with no side effects is critical. There is a scarcity of research on the genus *Barleria* exploring the antihelmintic activity of its plant extracts; Table 10 displays the few studies investigating this activity reported in the literature. Chander et al. [258] examined the antihelmintic activity of *B. buxifolia* water and ethanolic leaf extracts against *Pheretima posthuman* worms. They found that the ethanolic extract at 100 mg/mL produced a significant effect (*p* < 0.001) compared with the water extract. The water extract caused a dose-dependent paralysis that varied from loss of motility, loss of response to stimuli, and ultimately progressed to death. In the *P. posthuma* worms, the ethanolic extract took 37.75 ± 2.06 min for paralysis and 89.00 ± 1.82 min for death, whereas the duration for the water extract was 64.00 ± 2.16 min for paralysis and 150.50 ± 2.64 min for death. Chavan et al. [259] also evaluated the antihelmintic activity (paralysis and time of death) of whole water and ethanolic extracts of *B. prionitis* against *P. posthuma*. They reported that both water and ethanolic extracts significantly demonstrated paralysis (*p* < 0.01) in worms at lower doses (50, 75, and 100 mg/mL) and resulted in death at a high concentration of 100 mg/mL compared with albenadazole (standard).

### 3.12. Antiarthritic Activity

Rheumatoid arthritis is an autoimmune disease characterized by synovial membrane inflammation, pain, peripheral joint inflammation, destruction of articular tissue, and joint movement restriction [262,263,264]. This disease can affect an individual’s ability to perform daily tasks and causes premature death [265]. Irrespective of the progress made in the management of this disease, the treatments fail to generate long-term benefits, thus resulting in adverse effects such as renal morbidity, gastrointestinal ulcers, hematological toxicity, and cardiovascular complications [266,267]. This necessitates identifying alternative methods that cause less to no adverse effects. Therefore, it is essential to explore drugs from plant sources that exhibit antiarthritic activity. Table 11 summarizes the reported antiarthritic properties of various extracts and fractions of *Barleria*. A study conducted by Choudhary et al. [268] investigated the antiarthritic potential of ethyl acetate fractions from the leaves of *B. prionitis* against Freund’s complete adjuvant-induced chronic arthritis and formaldehyde-induced acute nonimmunological arthritis in rats. They reported significant inhibition of edema in Sprague Dawley rats in acute and chronic models. Diclofenac sodium served as the positive control in this study. The fraction used at a dose of 250 mg/kg exhibited potent and significant (*p* ≤ 0.05–0.01) inhibition of paw edema. Ethyl acetate fraction was found to decrease the histopathological changes induced by Freund’s complete adjuvant. Further studies are required to carry out the isolation of active constituents of the fraction responsible for the above activity. Overall, their study results disclosed the potential use of *B. prionitis* fraction in protecting the synovial membrane through hematinic parameters, thus demonstrating promising antiarthritic activity.

### 3.13. Antihypertensive Activity

Hypertension, also defined as high blood pressure, is an ailment in which blood vessels persistently increase the blood pressure of an individual [272]. This ailment contributes to the burden of premature mortality, heart diseases, disability, stroke, and kidney failure. Although several conventional antihypertensive drugs are used for hypertension treatment, they have adverse side effects such as extreme tiredness, dizziness, cramps, dehydration, and abnormal heart rate [273]. Therefore, researchers are focusing on herbal drugs as a source of treatment. Moreover, it is important to examine plants and their derivatives for antihypertensive activity. In this context, the methanolic leaf extracts of *B. prionitis* at doses of 200 and 400 mg/kg were found to exhibit antihypertensive effects and displayed 103 ± 2.54, 100.5 ± 2.74, and 105.5 ± 2.35 mm Hg of diastolic blood pressure and 136.5 ± 2.51, 146 ± 2.21, and 143 ± 3.11 mm Hg of systolic blood pressure after a 6-week treatment period [274].

### 3.14. Antiviral Activity

Viral infections are the primary causes of diseases because of their complexity and diversity. This makes it difficult to counteract their diffusion and effects, which often result in pandemic events [275]. Moreover, the increased frequency of global travel, urbanization, and migration have rendered virus outbreaks a challenging issue for public health, specifically when antiviral therapies and vaccines are not available [276]. In addition, the unsuccessful rate of numerous conventional drugs against viral infections and the onset of viral resistances have resulted in a growing interest in plants for promising antiviral agents [277]. Yoosook et al. [58] analyzed the leaf extracts of *B. lupulina* for intracellular activities against HSV-2 and five clinical HSV-2 isolates. Acyclovir was used as a positive control for anti-HSV in this study. Their study results demonstrated that the extracts exhibited activity against all the five clinical HSV-2 isolates. Further studies should be conducted using different assays on clinical isolates and not only standard strains of the virus. Chen et al. [87] also reported about the isolation of iridoid glycosides (6-*O*-*trans*-*p*-coumaroyl-8-*O*-acetylshanzhiside methyl ester and its *cis* isomer) from the methanolic extracts of *B. prionitis*, and these extracts were found to exhibit potent in vitro activity against the respiratory syncytial virus (EC_50_ 2.46 μg/mL, IC_50_ 42.2 μg/mL) [54].

### 3.15. Inhibition of Acetylcholinesterase Activity

Acetylcholine is a neurotransmitter at all parasympathetic, preganglionic autonomic, and sympathetic postganglionic nerve endings, as well as at the neuromuscular junction and at some central nervous system synapses. Acetylcholinesterase (AchE) inhibitors comprise several compounds of diverse structures and have the ability to inhibit the acetylcholine neurotransmitter [278,279]. AchE inhibitors are the most common drugs used in the treatment of diseases such as Parkinson’s, Alzheimer’s, senile dementia, and ataxia [280]. However, drugs such as rivastigmine, galantamine, and donepezil have limitations for medical use due to their adverse side effects [281]. Therefore, it is necessary to explore the plant kingdom for drugs that may inhibit acetylcholinesterase. The various extracts and isolated compounds of *Barleria* with reported acetylcholinesterase inhibitory activity are summarized in Table 12.

Amoo et al. [98] evaluated the acetylcholinesterase inhibitory activity of the methanolic extract of *B. prionitis* and found that it exhibited a dose-dependent inhibition action. The positive control used in this study was galanthamine. The AChE inhibition activities by galanthamine at 0.5, 1.0 and 2 μM were 49.24, 59.81 and 77.03%, respectively. At a higher concentration of extract (625 μg/mL), the leaf and stem of *B. prionitis* demonstrated greater inhibitory activity than its root extract. Kosmulalage et al. [86] also reported about the isolation of various compounds from the ethanolic extract of *B. prionitis* and their potential in inhibiting acetylcholinesterase. Balarenone, along with lupeol, pipataline, and 13,14-seco-stigmasta-5,14-diene-3-α-ol, isolated from ethanolic extract demonstrated moderate inhibitory activity against AChE [86]. Three distinct derivatives of pipataline, viz., 8-amino-7-hydroxypipataline, 7,8-epoxypipataline, and 7,8-dibromopipataline, were further synthesized to evaluate their inhibitory potential against acetylcholinesterase. Among the tested compounds, the best results were observed with 8-amino-7-hydroxypipataline, which exhibited significant acetylcholinesterase inhibitory activity with an IC_50_ value of 36.8 μM. Therefore, plant species within the genus *Barleria* demonstrate significant potential in inhibiting acetylcholinesterase activity.

### 3.16. Toxicology/Safety of Extracts of Barleria

Narmadha and Devaki [282] evaluated the acute toxicity and effective dose determination of the ethanolic leaf extract of *B. cristata* L. in wistar albino rats. Based on their body weight (250, 500, 1000 and 2,000 mg/kg), the ethanolic leaf extract were administered orally as a single dose to rats. Results showed that the administration of the ethanolic leaf extract at all doses (up to 2000 mg kg) did not produce any sign of acute toxicity or instant death in rats while tested during the period of observation. Singh et al. [240] evaluated the induced hepatotoxicity of the ethanol-water extract of the leaves and stems of *B. prionitis* in various experimental models, CCl_4_, D-GalN and paracetamol. In the safety evaluation study the oral LD_50_ was found to be >3000 mg/kg, with no signs of mortality after a single dose of drug administration. Kumari et al. [42] determined the toxicity of the methanol leaf and stem extracts by selecting different concentration of doses administered to albino rat (% mortality by using standard test). No mortality of albino rats (200, 400 and 600 mg/kg body weight) was recorded in both treatments of extracts. There is a scarcity of information on the toxicology and safety of extracts of *Barleria*, thus further studies are required.

## 4. Synthesis of Silver Nanoparticles from Plant Extracts of Species within *Barleria*

Nanotechnology is an emerging field that focuses on the synthesis and application of small materials known as nanoparticles (<100 nm) [283,284,285,286,287]. The physical properties of nanoparticles such as their size, shape, morphology, and their large surface-area-to-volume ratio have optimized their activity in various fields such as chemistry, medicine, and agriculture [288,289,290]. Significant development has been made in the study of metal-derived nanomaterials for their therapeutic and biomedical applications [291]. The development of multiple drug-resistant microorganisms poses a worldwide threat to public health [292]. Inappropriate use of antibiotics allow microorganisms to develop mutations, thereby making them resistant to conventional biocides [285,292,293]. Treatment of diseases caused by drug-resistant pathogens can lead to increased rates of morbidity and mortality [294,295,296]. Therefore, there is a need for extensive research in nanotechnology for identifying an effective treatment against drug-resistant bacteria [297].

Synthesis of nanoparticles from plants has received considerable attention due to their efficient use as reducing and capping agents of metals and their broad range of pharmacological applications [298]. Plants are widely available and less toxic, making this technique environmentally friendly and cost effective [299,300]. Medicinal plants are an abundant source of biologically active compounds. It is assumed that the bioreduction of nanoparticles using plant extracts is merely due to the presence of phytochemicals such as flavones, organic acids, polyphenols, and quinones [301,302]. The most frequently used metal nanoparticle for synthesizing plant constituents is silver [303]. Silver nanoparticles (AgNPs) are extremely toxic to multidrug-resistant bacteria [304].

Table 13 summarizes the biological activities of synthesized AgNPs from various extracts of *Barleria*. Govindarajan and Benelli [305] examined the toxicity of AgNPs synthesized from *B. cristata* leaf extracts against the larvae of *Aedes albopictus* (LC_50_ value 12.46 μg/mL), *Culex tritaeniorhynchus* (LC_50_ value 13.49 μg/mL), and *Anopheles subpictus* (LC_50_ value 15.01 μg/mL) (vectors of mosquitoes). The synthesized AgNPs demonstrated acute toxicity at low dosages against the various larvae of mosquitoes [305]. Overall, their study results emphasized that AgNPs synthesized from *B. cristata* are promising and ecofriendly agents that can be used against the vectors of mosquito. In addition, Gomathi et al. [306] reported that AgNPs synthesized from the leaf extracts of *B. cristata* exhibited potent antimicrobial activity. The nanoparticles demonstrated extremely promising antibacterial activity against *E. coli* and *S. aureus* that were inhibited considerably [306]. These studies have shown that the phytochemical compounds present in leaf extracts could serve as reducing and capping agents of silver nitrate (AgNO_3_), a frequently used precursor in AgNP synthesis. Medicinal plants are considered as a promising biological route for the synthesis of biocompatible metal nanoparticles. There is a scarcity of scientific information on the synthesis of AgNPs from plants extracts of species within *Barleria*. Therefore, it is necessary to screen more plant extracts for the biosynthesis of AgNPs as these particles have promising use in the nanotechnology industry and can be used as an affordable, environmentally friendly alternative to conventional medicine.

## 5. Advantages and Challenges

To our knowledge, this review represents the first detailed report summarizing the phytochemical analysis of species belonging to the genus *Barleria* and correlating the pharmacological effects with its most important compounds. Information from this review combines reported literature on various species within *Barleria*, thus providing baseline information on the potential usage of the extracts. This review may offer as a model for studies trying to scientifically explain medicinal plants effects. Traditionally, the genus *Barleria* has significant medicinal potential; however, there is a scarcity of information on the clinical and food applications on species within the genus and a lack of scientific information on the biological activities of several species. The safety efficacy of several plants are not documented and need to be further validated. These are all aspects which deserve further validation.

## 6. Conclusions

This review describes a comprehensive account of the phytochemical constituents and biological activities of plants belonging to the genus *Barleria*. Several bioactive compounds isolated from *Barleria* species, such as iridoids, phenolics, flavonoids, terpenoids, phytosterols and phenylethanoid glycosides possess various biological properties of medicinal importance. Moreover, both extracts and bioactive compounds from *Barleria* have demonstrated several biological activities, including antioxidant, antibacterial, antifungal, anti-inflammatory, anticancer, antidiabetic, antiulcer, hepatoprotective, analgesic, antiamoebic, antihelmintic, antiarthritic, antihypertensive, antiviral, and acetylcholinesterase activity inhibition properties and the ability to synthesize silver nanoparticles. Further investigations are recommended to explore more about the species within *Barleria* to identify new therapeutic compounds or drug leads, as most of them have not yet been subjected to chemical and biological assessment. Therefore, further research on the bioactive compounds and pharmacological activities of plants within this genus will provide a basic understanding of the importance of these species as medicinal plants and a potential source of novel and useful drugs.

## Figures and Tables

**Table 1:** Chemical compounds isolated from the Genus *Barleria*.

Plant Species	Plant Part	Chemical Group	Chemical Compounds/Phytoconstituents	Reference
*B. acanthoides*	Whole	Phenolic glycosides	Barlerisides A	[72]
			Barlerisides B	
		Phenylethanoid glycoside	Verbascoside (acteoside)	
		Phenolic acid	*p*-hydroxycinnamic acid	
		Neolignan diglycoside	Barlericin	[73]
*B. cristata*	Whole	Phenolic acid	4-hydroxy-*trans*-cinnamate	[70]
		Terpenoid	oleanolic acid	
	Bark	Flavonoid	6-*O*-α-L-rhamnopyranoside-3,7,3′-*O*-trimethylated-8-hydroxyquercetin	[74]
			6-*O*-α-L-rhamnopyranoside quercetagetin	
			3-*O*-Methylquercetin	
			Gossypetin 8-methyl ether	
			Quercetagetin	
			Tamarixetin	
			Gossypetin	
			Quercetin	
	Leaves	Phenolic acids	p-Coumaric acid	[75]
		Lipid	α -Tocopherol	
		Flavonoid	Luteolin	
		Flavonoid	7-*O*-Methylluteolin	
		Iridoid glycosides	Barlerin	
			Shanzhiside methyl ester	
		Phenylethanoid glycosides	Desrhamnosyl acteoside	[76]
			Poliumoside	
			Acteoside (verbascoside)	
*B. dinteri*	Leaves	Iridoid glycosides	Barlerin	[77]
*B. lupulina*	Aerial		8-*O*-acetylipolamiidic acid	[78,79,80,81,82,83,84]
			8-*O*-acetyl-6-*O*-(*p*-methoxy-*cis*-cinnamoyl)shanzhiside	
			8-*O*-acetyl-6-*O*-(*p*-methoxy-transcinnamoyl) shanzhiside	
			6-*O*-*p*-methoxy-*cis*-cinnamoyl-8-*O*-acetylshanzhiside methyl ester	
			6-*O*-*p*-methoxy-*trans*-cinnamoyl-8-*O*-acetylshanzhiside methyl ester	
			6-*O*-*p*-*cis*-coumaroyl-8-*O*-acetylshanzhiside methyl ester	
			6-*O*-*p*-*trans*-coumaroyl-8-*O*-acetylshanzhiside methyl ester	
			Ipolamiide	
			Ipolamiidoside	
			Shanzhiside	
			Shanzhiside methyl ester	
			8-*O*-acetylshanzhiside	
			Barlerin	
			6-*O*-acetylshanzhiside methyl ester	
			Acetylbarlerin	
			Mussaenosidic acid	
			Phlorigidoside	
		Iridoid diglucoside	Lupulinoside	
		Phenylethanoid glycosides	Forsythoside	
			Poliumoside	
		Lignan glucosides	(+)-lyoniresinol 3 α-*O*- β-glucopyranoside	
		Glycoside lipid molecule	1-octen-3-yl- β -primeveroside	
		Glycoside	Benzyl β -primeveroside	
*B. noctiflora*	Leaves	Phenylethanoid glycoside	Barlerinoside	[85]
*B. prionitis*	Aerial	Terpenoid	Balarenone	[86]
		Phenylethanoid glycoside	Barlerinoside	[67]
		Phenylethanoid glycoside	Verbascoside	[87]
		Iridoid glycosides	Barlerin	[65,67]
			Acetylbarlerin	
			Shanzhiside methyl ester	[87]
			6-*O*-trans-*p*-coumaroyl-8-*O*-acetylshanzhiside methyl ester	
			6-*O*-*cis*-coumaroyl-8-*O*-acetylshanzhiside methyl ester	
			7-methoxydiderroside	[67]
			Lupulinoside	
		Terpenoid	Pipataline	87]
			Lupeol	
		Phytosterols	13,14-seco-stigmasta-5,14-diene-3-β –ol	
	Roots		β-sitosterol	[88]
	Aerial	Flavonoid	Apigenin 7-*O*-β-D-glucoside	[78,86,89,90]
	Leaves		6-hydroxyflavone	[91]
			Scutellarin	
	Aerial		Luteolin-7-*O*-β-D-glucoside	[92]
	Leaves	Phenolic acid	Melilotic acid	[93]
			Syringic acid	[91]
			Vanillic acid	
			p-hydroxybenzoic acid	
*B. strigose* Whole		Phenylethanoid glycoside	4-hydroxyphenylethyl 4-*O*-β-D-glucopyranosyl-(1→3)-*O*-α-L-rhamnopyranoside	[94]
		Phenylethanoid glycoside	Verbascoside	
		Iridoid glycoside	10-*O*-trans-coumaroyl-eranthemoside	
			Decaffeoylverbascoside	
			Lyoniresinol 3 α -*O*-β -D-glucoside	
			7-*O*-acetyl-8-epi-loganic acid	
			(3R)-1-octen-3-ol-3-*O*-β-D-xylosyl-(1→6)-β-D-glucoside	
		Phenylethanoid glycosides	Isoverbascoside	
			Decaffeoylverbascoside	
		Flavonoid	Apigenin 7-*O*-α-Lrhamnosyl-(1→6)-*O*-β-D-glucoside	
*B. trispinosa*	Aerial	Iridoid glycosides	6-α-L-rhamnopyranosyl-8-*O*-acetylshanzihiside methyl ester	[95]
			Acetyl barlerin	
			Barlerin	
			Shanzhiside methyl ester	

Synonyms: Acteoside = verbascoside.

**Table 2:** Antioxidant properties of extracts and compounds isolated from *Barleria*.

Plant Species	Plant Part	Extract/Compound	Antioxidant Activity/Models/Assays	Reference
*B. acanthoides*	Whole	Barleriside A, barleriside B	Superoxide scavenging activity, Xanthine oxidase activity	[72]
*B. albostellata*	Leaves, stems	Methanol	DPPH assay; FRAP assay; β-Carotene-linoleic acid model system	[98]
*B. argillicola*	Whole	Methanol	β-Carotene–linoleic acid model system; DPPH assay	[142]
*B. courtrallica*	Leaves	Ethanol	DPPH assay, hydroxyl radical scavenging activity, superoxide radical scavenging activity, ABTS assay and reducing power methods.	[143]
*B. cristata*	Leaves	Ethanol	DPPH assay, Superoxide anion and nitric oxide radical scavenging activity, hydrogen peroxide scavenging activity	[144]
	Leaves	Ethanol	DPPH assay, ABTS assay, TPTZ assay	[145]
	Leaves	Acetone and methanol	DPPH assay; Nitric-oxide Reducing Assay, FRAP	[146]
	Leaves	Ethanol	DPPH assay, FRAP	[147]
*B. dinteri*	Leaves	Acetone and methanol	DPPH assay	[148]
*B. gibsoni*	Leaves	Ethanol	DPPH assay; Nitric oxide radical scavenging activity	[53]
*B. grandiflora*	Leaves	Water and ethanol	FTC method, TBA method	[149]
*B. greenii*	Leaves, stems, roots	Methanol	DPPH assay; FRAP assay; β-Carotene-linoleic acid model system	[98]
*B. lupulina*	Leaves, stems	Methanol	DPPH assay	[42]
*B. montana*	Leaves	Ethanol	DPPH, Reducing power assay, Nitric oxide scavenging activity	[150]
	Leaves	Methanol	DPPH assay, Hydrogen peroxide method	[151]
*B. mysorensis*	Leaves	Water	DPPH assay	[152]
*B. noctiflora*	Leaf, roots	Methanol	DPPH assay, Ferrous reducing power, Fe^2+^ reducing power, Fe^2+^ chelating activity assay, Nitric oxide scavenging activity, ABTS assay, Superoxide anion scavenging activity, Hydrogen peroxide radical scavenging activity	[153]
	Whole	Ethanol	DPPH assay	[154]
	Aerial	Ethanol and Water	DPPH assay, ABTS assay, Scavenging of hydrogen peroxide (H_2_O_2_), Lipid Peroxidation Inhibitory Activity, Hydroxyl radical scavenging activity p-NDA method, Superoxide radical scavenging activity by alkaline DMSO method	[155]
*B. prionitis*	Leaves, stems, roots	Methanol	DPPH assay; FRAP assay; β-Carotene-linoleic acid model system	[98]
	Aerial	Ethanol	β carotene bleaching assay, DPPH assay and hydroxyl radical scavenging activity	[140]
	Aerial	Shanzhiside methyl ester, 6-*O*-trans-*p*-coumaroyl-8-*O*-acetylshanzhiside methyl ester, barlerin, acetylbarlerin, 7-methoxydiderroside, lupulinoside	DPPH assay	[67]
	Whole	Ethyl acetate	DPPH assay	[156]
	Leaves, stems	Acetone	FRAP assay, DPPH assay, ABTS Assay	[157]
	Leaves, stems	Methanol	DPPH, Reducing power assay	[158]
	Bark, leaves	Methanol	DPPH assay	[159]
	Leaves	Ethanol	FTC method, TBA method, Scavenging of hydrogen peroxide radicals, DPPH assay	[160]
	Whole	Ethanol	DPPH, ABTS Assay, Hydroxyl radical scavenging activity, Reducing power assay, Nitrous oxide Reducing Assay	[69]
	Flower	Ethanol and water	DPPH assay	[161]
*B. strigosa*	Roots	Ethanol and water	ABTS assay, Nitric oxide quenching assay, Ferric reducing assay, DPPH,	[162]
	Leaves	Methanol	DPPH assay	[163]

DPPH—(1,1-Diphenyl-2-picrylhydrazyl) free radical-scavenging activity; FRAP—Ferric Reducing Antioxidant Power, ABTS—(2,2′-azino-bis(3-ethylbenzothiazoline-6-sulfonic acid)) free radical-scavenging activity; FTC—Ferric thiocyanate method; TBA—Thiobarbituric acid method; TPTZ—2,4,6-tripyridyl-s-triazine radical scavenging assay; TBA—Thiobarbituric acid method.

**Table 3:** Antibacterial activities of extracts from species within *Barleria*.

Plant Species	Plant Part	Extract	Antibacterial Activity	Agent Dosage (μg/mL)	Reference
*B. acuminata*	Leaves	Ethanol	*B. cereus, B. subtilis, E. faecalis, S. aureus, S. epidermidis, E. coli, K. pneumonia, P. mirabilis, S. typhi, S. dysentriae.*	50,000	[170]
*B. albostellata*	Leaves, stem	Petroleum ether, dichloromethane	*B. subtillis, S. aureus, E. coli, K. pneumoniae*	50,000	[66]
*B. argillicola*	Whole	Methanol	*E. coli, P. aeruginosa, S. aureus*	20,000	[142]
*B. cristata*	Bark	Ethanol	*S. aureus, B. subtillis, S. mutans*	10,000	[74]
	Leaves	Methanol	*K. pneumonia, S. aureus, E. coli, S. paratyphi*	5000	[144]
		Methanol and water	*S. pyogenes, E. coli*	300	[171]
		Petroleum ether, chloroform and water	*X. oryzae, B. subtilis, E. coli, P. aeruginosa, P. fluorescences.*	-	[172]
*B. dinteri*	Leaves	n-hexane, dichloromethane, acetone and methanol	*E. coli, E. faecaelis, S. aureus, P. aeruginosa.*	10,000	[148]
*B. grandiflora*	Aerial	Ethanol	*S. aureus; S. mutans*	5000	[173]
*B. greenii*	Stems, roots	Dichloromethane	*B. subtillis, S. aureus, E. coli, K. pneumoniae*	30,000	[66]
*B. lupulina*	Whole	Methanol	*Propionibacterium acnes*	-	[64]
	leaves	Methanol	*S. aureus, E. coli; P. aeruginosa, K. pneumoniae, S. typhi*	200,000	[42]
	Leaves, stems	Ethanol	*S. aureus, E. coli, P. aeruginosa, K. pneumonia, S. typhi*	20,000	[174]
	Leaves	Methanol	*S. aureus, B. pumilus*	1250	[175]
	Leaves	Essential oil	*B. pumilus, S. aureus*	-	[176]
*B. montana*	Leaves	Acetone	*E. coli; S. typhi; P. aeruginosa; K. pneumoniae; P. vulgaris; B. subtilis; S. pneumoniae; S. aureus; E.coli*	200	[177]
	Aerial	Methanol	*B. subtilis; B. cereus; B. pumilis; S. aureus;* *E. coli; P. aeuriginosa; P. vulgaris; S. marceseans*	200,000	[178]
	Leaves	Water, ethanol, methanol, chloroform	*E. aerogenes; E. coli; S. pneumoniae; B.subtilis; P. vulgaris*	100	[179]
*B. prionitis*	Leaves, stems	Dichloromethane	*B. subtilis, S. aureus, E. coli, K. pneumoniae*	50,000	[66]
	Bark	Methanol	*S. mutants, S. aureus, Pseudomonas sp., Bacillus sp.*	50,000	[169]
	Leaves	Chloroform	*S. typhi; B. subtilis; V. cholera; M. luteus; Providencia sp.; L. sporogenus, Citrobacter sp.*	50,000	[180]
	Leaves	Water, petroleum ether, chloroform, acetone	*L. rhamnosus*	200,000	[181]
	Leaves	Ethanol	*S. typhi; B. subtilis;* *S. aureus; V. cholera; E. coli*	10,000	[182]
	Leaves, stem	Ethyl acetate	*B. pumilus; B. subtilis; S. pyogenes; B. cereus; S. marcescens, C. acidovorans; P. aeruginosa*	100,000	[183]
	Leaves	Methanol	*S. mutants; S. aureus; L. acidophilus; Pseudomonas sp.*	10,000	[184]
	Leaves	Petroleum ether, chloroform, water	*B. subtilis; E. coli; P. fluorescens; X. oryzae*	-	[172]
	Leaves	Ethanol	*S. aureus; B. subtilis; P. vulgaris; K. pneumonia; E. coli; P. aeruginosa*	10,000	[185]
	Aerial	Ethanol	*B. cereus; P. aeruginosa*	-	[86]
*B. strigosa*	Leaves	Butanol	*B. subtilis; S. aureus; M. luteus*	2000	[186]

*Bacillus cereus; Bacillus pumilus; Bacillus subtilis; Bacillus species.; Comomonas acidovorans; Citrobacter species; Enterobacter aerogenes, Enterococcus faecalis; Escherichia coli; Klebsiella pneumoniae; Lactobacillus acidophilus; Lactobacillus rhamnosus; Lactobacillus sporogenus; Micrococcus luteus; Proteus mirabilis; Proteus vulgaris; Pseudomonas aeruginosa; Pseudomonas fluorescences; Pseudomonas sp.; Psuedomonas vulgaris; Salmonella paratyphi; Salmonella typhi; Serratia marceseans; Shigella dysentriae; Staphylococcus aureus; Staphylococcus epidermidis; Streptococcus mutants; Streptococcus pneumoniae; Streptococcus pyogenes; Streptococcus species; Vibrio cholera; Xanthomonas oryzae.*

**Table 4:** Antifungal activities of extracts from species within *Barleria*.

Plant Species	Plant Part	Extract	Antifungal Activity	Agent Dosage (μg/mL)	Reference
*B. albostellata*	Leaves and stems	Petroleum ether, dichloromethane	*C. albicans*	20,000	[98]
*B. cristata*	Leaves	Saponin fraction	*C. albicans; A. flavous; Penicillium sp.; A. niger; Trichophyton sp.*	1000	[192]
		Saponin fraction	*A. flavous; A. niger*	5000	[144]
		Petroleum ether, chloroform, water	*A. flavous; C. albicans*	-	[172]
*B. grandiflora*	Leaves	Water	*A. fumigatus*	625	[193]
	Aerial	Ethanol	*C. albicans*	5000	[173]
	Leaves	Ethanol	*C. albicans*	1600	[194]
*B. greenii*	Leaves, stems and roots	Dichloromethane	*C. albicans*	20,000	[98]
*B. montana*	Aerial	Methanol	*A. niger; R. stolonifera; S. cerevisiae; P. chrysogenum*	200,000	[178]
*B. prionitis*	Bark	Methanol	*S. cerevisiae; C. albicans*	50,000	[169]
	Roots and stems	Petroleum ether, dichloromethane	*C. albicans*	20,000	[98]
	Leaves, stems and roots	Ethanol	*A. fumigatus; C. vaginitis; C. neoformans; C. albicans; B. dermatitidis*	20,000	[195]
	Aerial	Ethanol	*C. albicans*	5000	[173]
	Leaves	Ethanol	*C. albicans*	1600	[194]
	Aerial	Methanol	*C. albicans*	200	[196]
		Methanol	*C. albicans, A. niger*	200,000	[197]

*Aspergillus flavous; Aspergillus fumigatus; Aspergillus niger; Blastomyces dermatitidis; Candida albicans; Candidal vaginitis; Cryptococcus neoformans; Pencillium chrysogenum; Penicillium* species; *Rhizopus stolonifera; Saccharomyces cerevisiae; Trichophyton species.*

**Table 5:** Anti-inflammatory activities of extracts, fractions and isolated compounds from species within *Barleria*.

Plant Species	Plant Part	Extract	Anti-Inflammatory Activity/Assays/Model	Agent Dosage	Reference
*B. albostellata*	Leaves and stems	Petroleum ether, dichloromethane, ethanol	COX-1, COX-2	0.25 μg/μL	[66]
*B. cristata*	Leaves	Water	CIO in rat paws, prostaglandins inhibitory activity, and acetic acid induced capillary permeability in mice.	500 mg/kg	[203]
		Methanol	Inhibited oedema produced by histamine and serotonin in rats. Reduction in the increased peritoneal vascular permeability in mice	500 mg/kg	[204]
*B. greenii*	Stems and roots	Petroleum ether, dichloromethane, ethanol	COX-1, COX-2	0.25 μg/μL	[66]
*B. lupulina*	Aerial	Water	Activated the Nrf2 cell defense pathway in human dermal microvascular endothelial cells	-	[205]
	Aerial	Methanol	Acute and sub-acute inflammation models of albino rats.	300 mg/kg	[63]
	Whole	Methanol, acetone	CIO in rat paws and ethyl phenylpropiolate-induced ear oedema in rats.	50–200 mg/kg	[206]
*B. montana*	Leaves	Ethanol	Formalin induced inflammation in male albino wistar rats.	300 mg/kg	[207]
*B. prionitis*	Leaves, stems and roots	Petroleum ether, dichloromethane, ethanol	COX-1, COX-2 assays	0.25 μg/μL	[66]
	Whole	Methanol-aqueous fractions (TAF)	CIO in adrenalectomised rats, activity in acute inflammation induced by carrageenan, histamine and dextran in rats	100 mg/kg	[202]
	Roots	Water fractions	CIO in rat paw model	400 mg/kg	[208]
	Aerial	Shanzhiside methylester, 8-*O*-acetyl shanzhiside methyl ester, iridoid glycosides, monoterpenoidal fraction	Stimulated rat neutrophils by inhibiting MPO, elastase and MMP-9 enzymes	10 μg/mL	[209]

Cyclooxygenase (COX); Carrageenan-induced oedema (CIO); Nuclear factor erythroid 2–related factor 2 (Nrf2); Matrix Metalloproteinase-9 (MMP-9); Myeloperoxidase (MPO).

**Table 6:** Anticancer activities of extracts and isolated compounds from species within *Barleria*.

Plant Species	Plant Part	Extract/Compounds	Assays/Cell Lines	Agent Dosage	Reference
*B. cristata*	Aerial	Isoverbascoside	NQO1 assay, murine hepatoma cell line Hepa-1c1c7	3.125 μM	[211]
	Leaves and bark	Methanol	Brine shrimp lethality assay, brine shrimp cysts	200 μg/mL	[146]
*B. gibsoni*	Leaves	Petroleum ether, chloroform	SRB assay, MDA, MB 4355 (Human breast cancer) and Hep G2 (Liver cancer cell line)	50 µg/mL	[213]
*B. grandiflora*	Leaves	Alcoholic	A-549 (human lung cancer) cells, DLA tumour cells and Vero (African green monkey kidney) normal cells	300 μg/mL	[212]
		Ethanol	MTT assay; Human gingival fibroblast cell lines, human dermal fibroblast cell lines	1000 μg/mL	[173]
*B. lupulina*	Leaves	Ethanol	MTT assay; cancerous THP-1 cell lines	100 μg/mL	[214]
		Ethanol	MTT assay; HepG2 cells	1000 μg/mL	[174]
*B. prionitis*	Leaves	Ethanol	MTT assay; Human gingival fibroblast cell lines, human dermal fibroblast cell lines,	1000 μg/mL	[173]
		Ethanol	SRB assay, breast (MCF-7), colon (DLD-1), lung (A549), breast metastatic (MDMAMB-468), lung metastatic (NCIH358) and colon metastatic (SW620)	100 μg/mL	[215]
*B. strigosa*	Leaves	Butanol	MTT colorimetric assay, Human hepatocellular carcinoma (HepG2), human breast adenocarcinoma (MCF7), human oral epidermoid carcinoma (KB), human colon adenocarcinoma (HT29), murine lymphocytic leukemia (P388), human cervical carcinoma (HeLa) as well as two normal cell lines including African green monkey kidney (Vero) and mouse subcutaneous connective tissue (L929)	2000 μg/mL	[186]

MTT—(3-(4, 5-dimethylthiazolyl-2)-2, 5-diphenyltetrazolium bromide); DLA—Dalton’s lymphoma Ascites; NQO1- (NAD(P)H dehydrogenase [quinone] 1); SRB—Sulphorhodamine B.

**Table 7:** Antidiabetic activities of extracts and fractions from species within *Barleria*.

Plant Species	Plant Part	Extract	Antdiabetic Activity/Assays/Models	Agent Dosage	Reference
B. bispinosa	Aerial	Methanol	Male Wister rats, Streptozotocin induced diabetic rats	500 mg/kg	[220]
B. cristata	Seeds	Ethanol	Wistar rats, alloxan-induced diabetic rats	200 mg/kg	[218]
	Leaves and roots	Ethanol and petroleum ether	Inhibition of alpha-amylase enzyme assay, Inhibition of alpha-glucosidase enzyme assay	-	[147]
B. lupulina	Aerial	Methanol	Male Wister rats, Streptozotocin-diabetic rats	300 mg/kg	[61]
B. montana	Aerial	Methanol	Wistar albino rats; Streptozotocin induced diabetic rats	400 mg/kg	[221]
B. noctiflora	Aerial	Ethyl acetate	Wister rats, Streptozotocin induced type-2 diabetes in rats	400 mg/kg	[222]
	Whole	Ethanol	In-vitro anti-diabetic activity was determined by inhibition of α-glucosidase and inhibition of α-amylase studies	500 μg/mL	[223]
	Aerial	Ethyl acetate	Wister rats, Streptozotocin induced diabetic rats	-	[224]
B. prionitis	Leaves and roots	Ethanol	Adult Albino rats, alloxan-induced diabetic rats	200 mg/kg	[219]
	Leaves, stems and roots	Alcohol	Albino rats, alloxan-induced hyperglycemic rats	200 mg/kg	[225]

**Table 8:** Antiulcer activities of extracts and fractions from species within *Barleria*.

Plant Species	Plant Part	Extract	Antiulcer Activity/Gastric Cytoprotective Activity/Models	Agent Dosage	Reference
*B. buxifolia*	Whole	Methanol	Wistar rats, PL and aspirin induced ulcers	400 mg/kg	[229]
*B. gibsoni*	Leaves	Ethanol	Wistar rats, PL-induced ulcer models	500 mg/kg	[53]
*B. lupulina*	Aerial	Methanol	Albino (Wistar) rats, PL ulceration in rats, stress-induced ulceration, drug-induced gastric ulcer in rats, duodenal ulcers in rats.	200 mg/kg	[62]
*B. prionitis*	Leaves	Methanol	Wistar rats; ethanol induced gastric mucosal lesions, indomethacin induced ulcer models	500 mg/kg	[52]
		Ethanol	Male Sprague–Dawley rats and female Swiss albino mice; PL- induced ulcers, aspirin- induced ulcers, CRS-induced ulcers, ethanol-induced ulcer	200 mg/kg	[228]
		Methanol	Ethanol and Indomethacin Induced ulcer models	500 mg/kg	[230]
		Chloroform	Rodent experimental models (indomethacin and pylorus ligation)	250 mg/kg	[52]

CRS—cold-restraint stress; PL—Pylorus ligated.

**Table 9:** Hepatoprotective activities of extracts and fractions from species within *Barleria*.

Plant Species	Plant Part	Extract	Hepatoprotective Activity/ASSAYS/Models	Agent Dosage (mg/kg)	Reference
*B. cristata*	Leaves	Ethanol	Wistar albino rats, CCl_4_ induced hepatic damage in rats	200	[238]
*B. cuspidata*	Leaves	Methanol	Wistar albino rats, CCl_4_ induced hepatotoxicity in rats	400	[241]
*B. gibsoni*	Aerial	Aqueous alcoholic	Wistar albino rats; inducing agent Paracetamol in Carboxy methyl cellulose	400	[242]
*B. montana*	Leaves	Methanol	Wistar albino rats; ethanol-induced rat hepatic injury	500	[243]
	Aerial	Methanol	CCl_4_ induced hepatotoxicity on rats	800	[178]
*B. prionitis*	Leaves and stems	Fractions from ethanol-aqueous	Charles Foster rats, Swiss albino mice; acute and chronic animal test models, CCl_4_ toxicity, cetaminophen (APAP) toxicity, D-GalN induced hepatotoxicity	200	[240]

CCl_4_—Carbon tetrachloride.

**Table 10:** Anthelmintic activities of extracts from species within *Barleria*.

Plant Species	Plant Part	Extract	Anthelmintic Activity/Assays/Models	Agent Dosage (mg/mL)	Reference
*B. buxifolia*	Leaves	Ethanol	IAW *P. posthuma*	100	[258]
*B. gibsoni*	Leaves	Water, ethanol	IAW *P. posthuma*	15	[260]
*B. prionitis*	Whole	Water, ethanol	IAW *P. posthuma*	100	[259]
		Water, ethanol	IAW *P. posthuma*	100	[261]

IAW—Indian adult worm; *Pheretima posthuma.*

**Table 11:** Antiarthritic activities of extracts and fractions from species within *Barleria*.

Plant Species	Plant Part	Extract	Antiarthritic Activity/Assays/Models	Agent Dosage (mg/kg)	Reference
*B. lupulina*	Leaves	Methanol	Albino male mice, female Sprague Dawley rats, formalin-induced arthritis, adjuvant induced arthritis, collagen type II-induced arthritis, monosodium iodoacetate induced osteoarthritis	600	[269]
*B. montana*	Leaves	Ethanol	Male Albino Wistar rats, Complete Freund’s in vivo method in induced rats	400	[270]
	Leaves	Ethyl acetate fraction	Sprague Dawley rats, formaldehyde induced arthritis; FCA-induced arthritis rat model	250	[268]
*B. prionitis*	Whole	Methanol	Complete Freund’s induced rat model	400	[271]

FCA—Freund’s complete adjuvant.

**Table 12:** Acetylcholinesterase inhibition of extracts and isolated compounds from species within *Barleria*.

Plant Species	Plant Part	Extract/Compound	Inhibition of Acetylcholinesterase/	Agent Dosage	Reference
*B. albostellata*	Leaves, stems and roots	Methanol	Microtitre plate assays based on the colorimetric method; and using the positive control galanthamine	625 μg/mL	[98]
*B. greenii*	Leaves, stems and roots	Methanol	Microtitre plate assays based on the colorimetric method; and using the positive control galanthamine	625 μg/mL	[98]
*B. prionitis*	Leaves, stems and roots	Methanol	Microtitre plate assays based on the colorimetric method; and using the positive control galanthamine	625 μg/mL	[98]
	Aerial	8-amino-7-hydroxypipataline	Modified Ellman’s assay, photometric method	-	[86]
		6-*O*-*trans*-*p*-coumaroyl-8-*O*-actylshanzhiside methyl ester, barlerin, acetylbarlerin, 7-methoydiderroside, lupulinoside	Ellman’s assay	-	[67]

**Table 13:** Biological activity of synthesized nanoparticles from extracts of species of *Barleria*.

Plant Species	Plant Part	Extract	Nanoparticles Synthesised	Reported Activity/Phytochemicals Present	Agent Dosage	Reference
*B. cristata*	Leaves	Water	Ag *	Mosquitocidal potential	300 μg/mL	[305]
			Ag	Antibacterial activity against *E. coli* and *S. aureus*	-	[306]
*B. longiflora*	Leaves	Water	Ag	Antimicrobial activity, inhibition of *Enterococcus sp.*, *Streptococcus sp*., *B. megaterium*, *P. putida*, *P. aeruginosa* and *S. aureus* and potential application in photocatalytic dye degradation processes	10 μg/mL	[307]
*B. prionitis*	Leaves	Water	Ag	Polyphenols, starch, reducing sugars, ascorbic acid and citric acid using GC-MS analysis	-	[308]

* Silver.

## Data Availability

Not applicable.
